# Integrative Transcriptomic Analysis Reveals Upregulated Apoptotic Signaling in Wound-Healing Pathway in Rat Liver Fibrosis Models

**DOI:** 10.3390/antiox12081588

**Published:** 2023-08-09

**Authors:** Jihyun Kim, Changyong Lee, Sang Gyun Noh, Seungwoo Kim, Hae Young Chung, Haeseung Lee, Jeon-Ok Moon

**Affiliations:** 1 BIT Convergence-Based Innovative Drug Development Targeting Mate-Inflammation, Pusan National University, Busan 46241, Republic of Korea; kimjh763@snu.ac.kr; 2 College of Pharmacy and Research Institute for Drug Development, Pusan National University, Busan 46241, Republic of Korea; qhrrn79@pusan.ac.kr (C.L.); rskrsk92@pusan.ac.kr (S.G.N.); nubakim@pusan.ac.kr (S.K.); hyjung@pusan.ac.kr (H.Y.C.)

**Keywords:** transcriptomic analysis, liver fibrosis, burn-wound healing, apoptosis, oligonol, hepatoprotective

## Abstract

Liver fibrosis, defined by the aberrant accumulation of extracellular matrix proteins in liver tissue due to chronic inflammation, represents a pressing global health issue. In this study, we investigated the transcriptomic signatures of three independent liver fibrosis models induced by bile duct ligation, carbon tetrachloride, and dimethylnitrosamine (DMN) to unravel the pathological mechanisms underlying hepatic fibrosis. We observed significant changes in gene expression linked to key characteristics of liver fibrosis, with a distinctive correlation to the burn-wound-healing pathway. Building on these transcriptomic insights, we further probed the p53 signaling pathways within the DMN-induced rat liver fibrosis model, utilizing western blot analysis. We observed a pronounced elevation in p53 protein levels and heightened ratios of BAX/BCL2, cleaved/pro-CASPASE-3, and cleaved/full length-PARP in the livers of DMN-exposed rats. Furthermore, we discovered that orally administering oligonol—a polyphenol, derived from lychee, with anti-oxidative properties—effectively countered the overexpressions of pivotal apoptotic genes within these fibrotic models. In conclusion, our findings offer an in-depth understanding of the molecular alterations contributing to liver fibrosis, spotlighting the essential role of the apoptosis pathway tied to the burn-wound-healing process. Most importantly, our research proposes that regulating this pathway, specifically the balance of apoptosis, could serve as a potential therapeutic approach for treating liver fibrosis.

## 1. Introduction

Liver fibrosis is a pathological condition characterized by the abnormal accumulation of extracellular matrix (ECM) proteins within liver tissue in response to chronic inflammation [1]. This process causes prolonged tissue scarring and dysfunction, accompanied by a wound-healing response. This condition activates hepatic stellate cells (HSCs) to produce large amounts of ECM proteins in the liver [2]. Apoptosis, a programmed cell death, plays a key role in wound healing by eliminating damaged cells; however, its dysregulation can lead to persistent inflammatory responses in wounds [3]. The normal balance of apoptosis can be disrupted by severe and repetitive injurious stimuli, resulting in the recruitment of inflammatory and immune cells and contributing to the development of fibrosis [4]. Additionally, excessive apoptosis has been implicated to contribute to liver fibrogenesis by promoting the activation of HSCs and myofibroblasts that produce ECM scars in the liver [5]. Prolonged liver injury can result in the development of liver cirrhosis, hepatocellular carcinoma (HCC), and ultimately liver failure [6]. 

The mechanisms implicated in the development of liver fibrosis have been widely investigated. Oxidative stress, including the formation of reactive oxygen species (ROSs), lipid peroxidation, and mitochondrial dysfunction, can cause the necrosis and apoptosis of hepatocytes [7]. Oxidative damage can also induce hepatic inflammation, apoptotic responses, and liver damage, which will further exacerbate the fibrotic response [8]. Animal models are indispensable tools to study the mechanisms underlying fibrogenesis and to evaluate the impact of anti-fibrotic therapies [9]. Various animal models have been developed for experimental liver fibrosis research, including hepatotoxin-induced, biliary, autoimmune, alcohol-induced liver disease, and non-alcoholic fatty liver disease [10]. Dimethylnitrosamine (DMN) and carbon tetrachloride (CCl_4_) are commonly used to induce liver fibrosis in rodents. Liver fibrosis is induced by DMN through the activation of HSCs and the enhancement of ROS production [11]. Liver fibrosis induced by CCl_4_ resembles properties of human pathologies, including inflammation and fiber formation, in rodents [12]. Bile duct ligation (BDL) is the archetype model to induce the rapid establishment of liver fibrosis in a specific context according to human pathology [13]. Although animal models have limitations due to species differences, they still provide valuable insights into the pathogeneses of diseases [14]. To extract relevant clues about pathogenesis, it is important to analyze multiple models induced by various factors and identify common robust gene signatures across the models, even for the same disease.

Various anti-fibrotic candidates have been identified for liver fibrosis through the use of anti-oxidants or blocking oxidative stress [15]. We have previously demonstrated that oligonol has a protective effect against oxidative stress by regulating the PI3K/AKT/NRF2 signaling pathway in DMN-induced liver fibrosis in rats [16]. Oligonol is a phenolic phytochemical consisting of catechin monomers, procyanidin dimers, and oligomers of proanthocyanidins [17]. Oligonol has exhibited strong anti-oxidant and anti-inflammation activities, serving as a promising candidate for the treatment of cancer as well as inflammatory and metabolic disorders [18,19]. Furthermore, oligonol possesses anti-aging properties that can prevent photoaging by mitigating changes that would cause dysregulation of wound healing, collagen synthesis, and growth factor production [20].

In this study, we took advantage of three independent liver fibrosis models and investigated their transcriptome data to understand the pathological mechanisms behind hepatic fibrosis. We compared the common gene signatures of liver fibrosis with those of oligonol-treated liver fibrosis and identified significant changes in apoptotic genes within the wound-healing pathway in liver fibrosis. 

## 2. Materials and Methods

### 2.1. Animal Model

In consideration of ethical and scientific care standards, the animal protocol implemented in this research was examined and approved by the PNU Institutional Animal Care and Use Committee (PNU-IACUC) under approval number PNU-2016-1417. Sprague-Dawley (SD) male rats, obtained from Samtako in Osan, Korea, were provided with standard rat food and unrestricted access to tap water while being maintained on a 12 h light–dark cycle. A total of twenty-four rats, aged 6–7 weeks and weighing 170–190 g, were allocated into four groups of six rats each: control, DMN, Oli10, and Oli20. The control group was given saline (serving as a DMN vehicle) through intraperitoneal (i.p.) injection and CMC (serving as an oligonol vehicle) via oral gavage. The DMN group was administered DMN and CMC, while the Oli10 and Oli20 groups received DMN in addition to oligonol at dosages of 10 and 20 mg/kg/day, respectively. To induce liver damage in the rats, i.p. injections of 1% (*w*/*v*) DMN (10 mg/kg body weight) were given daily for three consecutive days per week over a four-week duration. Oligonol was administered once daily for four weeks using oral gavage. After four weeks, all rats were sacrificed under anesthesia following an overnight fast. Blood samples for biochemical examination were taken from the inferior vena cava, and liver tissues were removed and instantly preserved in liquid nitrogen for further investigation.

### 2.2. Biochemical Analysis of Liver Enzymes

Serum aspartate transaminase (AST) and alanine transaminase (ALT) activities were measured using AST/ALT kits (Asan Pharm, Seoul, Republic of Korea) according to the method described by Reitman and Frankel [21].

### 2.3. RNA Extraction and RNA Sequencing (RNA-seq)

The total RNA was extracted from the liver tissues (0.1 g) using TRizol reagent (Invitrogen, Carlsbad, CA, USA), following the manufacturer’s protocol. The extracted RNA was assessed for purity and integrity using a BioDrop nano-spectrophotometer (Biochrom Ltd., Cambridge, UK). All samples showed high purity (optical densities (ODs): 260/OD280 > 1.8) and integrity (RNA integrity numbers > 7.0). For RNA-seq, cDNA libraries were constructed, and single-end libraries were sequenced using the MGI-T7 platform (MGI Tech Co., Shenzhen, China) and the MGIEasy RNA Directional Library Prep Set.

### 2.4. Collection of Public Transcriptome Data for Liver Fibrosis

We searched the Gene Expression Omnibus (GEO) for the keyword ‘liver fibrosis’ and obtained RNA-seq data sets obtained from the liver tissues of models of liver fibrosis induced by BDL (GSE152250) [22] and CCl_4_ (GSE189402) [23], respectively (Table 1). Raw fastq files were downloaded from the Sequence Read Archive (SRA) via the European Nucleotide Archive (https://www.ebi.ac.uk/ena/browser/, accessed on 6 April 2023). The metadata of the individual samples were obtained through the R package ‘GEOquery’.

### 2.5. Preprocessing of RNA Sequencing Data

The RNA-seq data sets analyzed in this study (Table 1) were preprocessed via the following protocol: First, adapter sequences and low-quality bases (Q scores < 20) were eliminated using TrimGalore (https://www.bioinformatics.babraham.ac.uk/, accessed on 5 September 2020). Next, cleaned reads were then mapped to the rat reference genome (mRatBN7.2) using the STAR aligner (v2.7.9a) [24] with the gene annotation mRatBN7 Gene Transfer Format file. Gene expression levels were finally quantified as expected read counts or transcript per million (TPM) values with RSEM software (v1.3.3).

### 2.6. Differential Expression and Functional Enrichment Analysis

The exact test implemented in the R package ‘edgeR’ was utilized to assess individual gene expression changes between two groups, such as liver fibrosis versus normal or oligonol-treated versus untreated. Differentially expressed genes (DEGs) were selected based on the following cut-offs: false discovery rate (FDR)-adjusted *p*-values < 0.01 and |log_2_fold-change| > log_2_(2). Functional overrepresentative analysis was performed via a hypergeometric test using the enricher function in the R package ‘clusterProfiler’ with Wikipathway and Gene Ontology (GO) [25] gene sets. The gene sets were downloaded from the Molecular Signature Database (MSigDB) using the R package ‘msigdbr’. The R package ‘fgsea’ [26] was further employed to perform Gene Set Enrichment Analysis (GSEA) in order to visually demonstrate the downregulation of the gene set ‘P53 transcriptional gene network’ in the DMN-induced rat liver fibrosis model following treatment with oligonol. Gene networks showing the gene–gene interactions involved in apoptosis and p53 signaling within the burn-wound-healing pathway were rendered using Cytoscape (v3.9.1).

### 2.7. Western Blot

Liver samples were homogenized in a ProEXTM CETi lysis buffer containing protease and phosphatase inhibitors (Translab, Daejeon, Republic of Korea) and then centrifuged at 12,000 rpm and 4 °C for 10 min. Protein concentrations were measured using the BCA assay, with BSA as a standard. Equivalent aliquots of protein samples (30 µg) were run on an SDS-polyacrylamide gel (8–12%) and transferred to a polyvinylidene difluoride membrane (Millipore, Billerica, MA, USA). The blots were then incubated in a blocking buffer containing 5% non-fat milk powder in tris-buffered saline Tween-20 (TBST) for 1 h, followed by primary antibodies in TBST overnight at 4 °C, with specific primary antibodies against CASPASE-3 (CASP3, sc-7148), β-actin (sc-47778) (Santa Cruz Biotechnology, Santa Cruz, CA, USA), p53 (#2524), BCL2 (#2870), BAX (#2772), poly-(ADP-ribose) polymerase (PARP) (#9532) (Cell Signaling Technology, Danvers, MA, USA), and GAPDH (GTX100118) (Gene Tex, Irvine, CA, USA) at a 1:1000 dilution. The membrane was washed with TBST and then incubated with 1:10,000 dilutions of anti-mouse and anti-rabbit secondary antibodies (Santa Cruz Biotechnology) for 1 h at room temperature. The blots were visualized with an ECL detection kit (Advansta, San Jose, CA, USA). The optical density of each band was quantified with ImageJ 1.53e software (NIH, Bethesda, MD, USA) and normalized to GAPDH as a loading control. 

### 2.8. Statistical Analysis

All data were analyzed and visualized using GraphPad Prism 5 (GraphPad Software, San Diego, CA, USA) or R software (v4.2.2). One-way analysis of variance followed by Tukey’s test was applied to assess the statistical significances of the mean differences between/among groups unless otherwise specified. A *p*-value of <0.05 was considered statistically significant unless otherwise noted.

## 3. Results

### 3.1. Common Transcriptomic Signatures of Multiple Liver Fibrosis Models Reveal Known Pathogenic Features of Fibrosis

**Figure 1:**
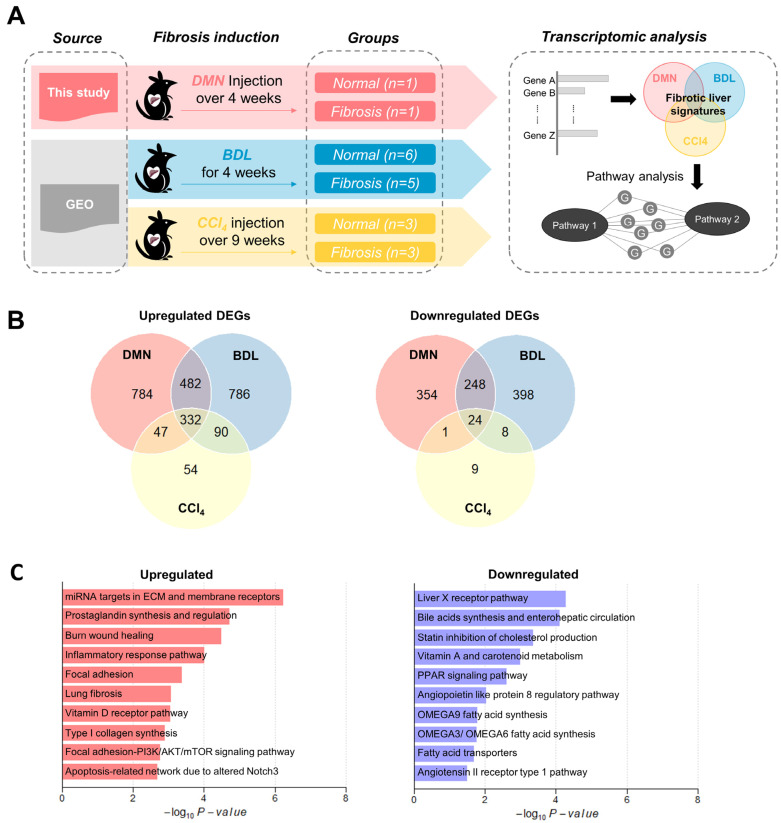
Common gene expression signatures among three liver fibrosis models. (**A**) Schematic diagram illustrating our transcriptomic analyses conducted on models of liver fibrosis induced by DMN, BDL, and CCl_4_. (**B**) Venn diagrams depicting the overlaps of DEGs across the three liver fibrosis models. (**C**) Top ten pathways significantly enriched in DEGs that were commonly upregulated or downregulated across all three fibrosis models. DMN, dimethylnitrosamine; BDL, bile duct ligation; CCl_4_, carbon tetrachloride.

We hypothesized that the genes involved in the pathogenesis of liver fibrosis change in expression as the disease progresses. To explore this, we investigated the liver transcriptome profiles of rat models with liver fibrosis induced by multiple stimuli (Figure 1A). We first generated DMN-induced fibrosis models and conducted RNA-seq analysis on lesional and normal liver tissues. To identify the robust gene expression signatures of liver fibrosis, we additionally collected publicly available RNA-seq data sets derived from two other models of liver fibrosis induced by BDL and CCl_4_, respectively (Table 1). We processed raw RNA-seq data from each model uniformly with the same pipeline and selected differentially expressed genes (DEGs) in the fibrotic liver tissue compared to in normal tissue (Supplementary Figure S1A and Supplementary Tables S1–S3). The common DEGs among the three models included 332 upregulated genes and 24 downregulated genes, showing that most of the altered genes exhibited increased expressions as the fibrosis advanced (Figure 1B). 

To identify the functional categories enriched among the common DEGs in all three models, we performed an overrepresentation analysis using the Wikipathway (Figure 1C and Supplementary Table S4) and GO gene sets (Supplementary Figure S1B). We found that gene signatures related to the typical fibrosis pathogenesis, which includes ECM remodeling, collagen synthesis, inflammatory responses, and prostaglandin regulation, were significantly upregulated in liver fibrotic tissue (Figure 1C and Supplementary Figure S1B). As fibrosis occurs as a result of chronic inflammation, it triggers the persistent activation of fibroblasts responsible for the synthesis and deposition of collagen fibers [27]. Consistently with this, previous studies have observed increased collagen synthesis in fibrosis models, along with the subsequent regulation of prostaglandin to inhibit collagen expression and deposition [28,29,30]. We observed an upregulation of the genes involved in the PI3K/AKT/mTOR signaling pathways, the dysregulation of which can drive the hyperactivation of inflammatory cells and HCC progression [31]. In addition, PI3K/AKT signaling is associated with the inhibitory effect of peroxisome proliferator-activated receptors (PPARs) on HSC activation [32]. 

Although the number of commonly downregulated DEGs was relatively low, they were enriched for several pathways, including the fatty acid metabolic process, PPAR signaling, and angiopoietin-like protein 8 regulatory pathways (Figure 1C and Supplementary Table S4). In particular, the liver X receptor (LXR) pathway, where LXR functions as a transcription factor that regulates the expressions of genes involved in lipid metabolism, including the synthesis of bile acids, cholesterol, and fatty acids, was the most significantly enriched pathway [33]. Previous studies have demonstrated that downregulation of the LXR pathway can lead to increased production of proinflammatory cytokines and chemokines [34], as well as the accumulation of lipids and ECM proteins [35]. PPARs are also known to regulate lipid metabolism, and their alpha and delta subfamilies have been shown to reduce inflammation, oxidative stress, and fibrosis by reducing lipid accumulation in the liver [36]. Furthermore, angiopoietin-like protein 8 plays a critical role in hepatic glucose metabolism, which is commonly impaired during liver fibrosis [37,38]. 

Overall, the fibrotic gene signatures derived from the transcriptomes of the three independent models revealed known molecular mechanisms associated with fibrosis, implying that these signatures could provide reliable evidence on the pathogenesis of liver fibrosis. 

### 3.2. Transcriptomic Signatures Indicate the Impact of Oligonol Treatment on the Burn-Wound-Healing Pathway in Liver Fibrosis Models

**Figure 2:**
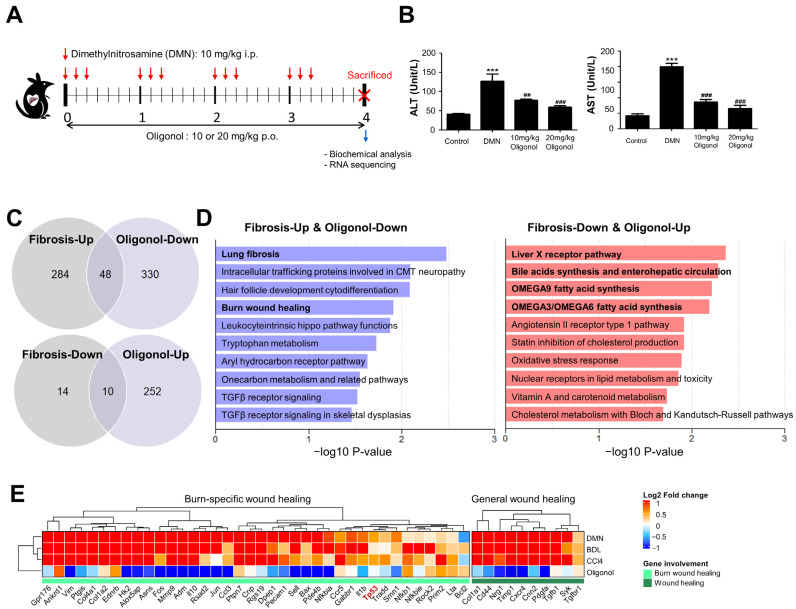
Impacts of oligonol on biochemical and transcriptome changes in liver fibrosis model. (**A**) Schematic representation of in vivo study design. The red arrows represent the time points at which DMN was administered, and the ‘X’ markings denote the time points at which the rats were sacrificed. (**B**) Alterations in serum ALT and AST levels. Bar plots show the mean values with the corresponding standard error of the mean (SEM) for each group (*n* = 6). Statistical significance is indicated as follows: *** *p* < 0.001 vs. the control group and ^##^ *p* < 0.01 and ^###^ *p* < 0.001 vs. the DMN group. (**C**) Venn diagrams illustrating the overlapping liver fibrotic signatures (common DEGs of three liver fibrosis models) and DEGs affected by oligonol treatment. (**D**) Top ten significantly enriched pathways for 48 genes upregulated in the DMN model and downregulated upon oligonol treatment (**left**) and 10 genes downregulated in the DMN model and upregulated upon oligonol treatment (**right**). (**E**) Expression changes (log2 fold-change) of genes involved in the burn-wound-healing pathways under different conditions. Genes exhibiting significant changes in at least two liver fibrotic models were selected. Highlighted in red is Tp53, a gene of particular interest in this study. DMN, dimethylnitrosamine; AST, aspartate transaminase; ALT, alanine transaminase; NESs, normalized enrichment scores.

In a previous study, we demonstrated that oligonol exhibits anti-inflammatory and protective effects against oxidative stress-induced liver fibrosis [16]. Therefore, we aimed to identify which fibrotic gene signatures are affected by oligonol treatment. To achieve this, the rats received i.p. injections of DMN once daily for three consecutive days per week for four weeks and were administered 10 or 20 mg/kg/day oligonol once daily for four weeks via oral gavage (Figure 2A). We measured serum levels of ALT and AST as indicators of liver injury and observed that DMN exposure increased these levels while oligonol treatment at both 10 and 20 mg/kg doses decreased them (Figure 2B). Subsequently, we conducted transcriptome profiling on the liver tissues from the DMN model, with oligonol administration at a dose of 20 mg/kg (Table 1). We identified the DEGs affected by the oligonol treatment by comparing the oligonol-treated DMN model with the untreated DMN model (Supplementary Figure S2A and Supplementary Table S5). Interestingly, among the downregulated DEGs affected by the oligonol treatment, 318 (84.12%) were upregulated in the DMN model, and among the upregulated DEGs affected by the oligonol treatment, 189 (72.13%) were downregulated in the DMN model (Supplementary Figure S2B). We further identified 48 downregulated DEGs and 10 upregulated DEGs with the oligonol treatment, which exhibited inverse regulation in all three liver fibrosis models (Figure 2C). Functional enrichment analysis on these genes revealed two overrepresented gene sets, lung fibrosis and burn-wound healing, which were the only gene sets significantly enriched in both the upregulated common DEGs of fibrosis (Figure 1C) and the downregulated DEGs of the oligonol treatment (Figure 2D). The burn-wound-healing pathway encompasses the known molecular players involved in the wound-healing response following burn injury in mammals [39]. The healing process for burn wounds typically involves three main phases, inflammation, proliferation, and remodeling, which parallels the responses to other wounds in the liver [39]. However, there are notable differences in terms of the molecular players, including the involvements of *p53*, *Bax*, *Mmp9*, and others (Supplementary Figure S2C). We focused particularly on the genes involved in the healing process specific to burn wounds and noted the significance of p53, as this aligns with the finding that the 318 genes that exhibited upregulation in the DMN-induced liver fibrosis model and downregulation upon oligonol treatment were significantly associated with the p53 transcriptional gene network (Supplementary Figure S2D). 

### 3.3. Expression of p53 Targets Was Overall Elevated in Liver Fibrosis Models

**Figure 3:**
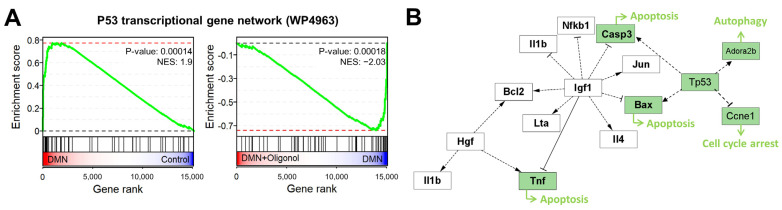
Association of p53 pathway and apoptotic genes with liver fibrosis. (**A**) GSEA plots displaying the upregulation of genes associated with the ‘P53 transcriptional gene network’ in liver tissues of the DMN group (*n* = 1) compared to the normal group (*n* = 1) and their downregulation in the oligonol-treated DMN group compared with the DMN group (*n* = 1). (**B**) A gene network representing gene–gene interaction associated with p53-related apoptotic genes in the burn-wound-healing pathway. Genes involved in both apoptosis and the p53 signaling pathway are colored green. DMN, dimethylnitrosamine; NESs, normalized enrichment scores.

The tumor suppressor protein p53 is a well-known transcription factor that regulates the expressions of a wide variety of genes involved in apoptosis and cell proliferation [40]. However, the specific role of p53 in liver fibrosis remains uncertain due to its complex interactions and context-dependent function [41]. Previous studies have shown that p53 activation leads to hepatocyte apoptosis, subsequent activation of HSCs, and the development of liver fibrosis [41,42]. Conversely, loss of p53 function or overexpression of its negative regulator MDM2 can enhance HSC activation and promote fibrosis progression [43]. As such, our understanding of p53’s role in liver fibrosis continues to evolve. In our liver fibrosis model, we observed a significant elevation of p53 downstream target gene expression levels in response to DMN, which were subsequently reduced by the oligonol treatment (Figure 3A). Additionally, we found strong positive correlations between the expression changes of these p53-associated genes in the DMN, BDL, and CCI_4_ models (Supplementary Figure S3). In contrast, these expression changes in the three models showed negative correlations with those in the oligonol-treated DMN model. These observations suggest that regulation of these p53-associated genes may serve as a plausible mechanism through which oligonol exerts its beneficial effects in liver fibrosis.

### 3.4. p53-Associated Apoptotic Genes May Be a Plausible Mechanism in Liver Fibrosis

**Figure 4:**
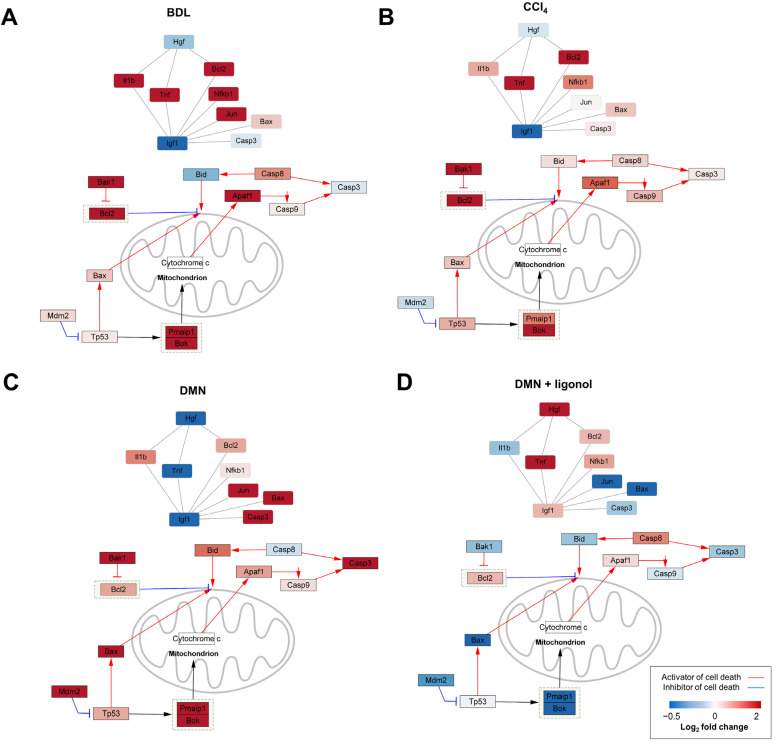
Transcriptomic changes in p53-associated apoptotic signaling in burn-wound-healing pathway. (**A**–**D**) Gene–gene network representing the relationships between genes involved in p53-induced apoptotic signaling in burn-wound-healing pathway. A node represents a gene and an edge represents an interaction between genes. Nodes are colored based on their expression changes (log2 fold change) following (**A**) BDL, (**B**) CCI_4_, (**C**) DMN, and (**D**) DMN + oligonol treatments.

Based on the elevated expression levels of the p53 targets in the liver fibrosis models and a previous report that p53 activation triggers the apoptotic pathways in hepatocytes [42], we further focused on the regulation of the p53-associated apoptotic genes, such as *Bax*, *Casp3*, and *Tnf*, that are involved in the burn-wound-healing pathway (Figure 3B). To explore whether this apoptotic pathway is associated with liver fibrogenesis, we examined the expression changes of the p53-associated apoptotic genes in the individual liver fibrosis models. We constructed a gene–gene network by integrating the p53 signaling, apoptosis, and burn-wound-healing pathways, where each node represented a gene and each edge represented the interaction between genes. These networks showed that at least two fibrosis inducers commonly upregulated the expression levels of *Tp53*, *Bax*, *Pmaip1*, *Box, Bak1*, *Casp3*, *Casp9*, *Bid*, *Il1b*, and *Jun* (Figure 4A–C), while oligonol downregulated their expression (Figure 4D). 

### 3.5. Apoptotic Genes Affected by DMN Exposure Are Restored with Oligonol Treatment

**Figure 5:**
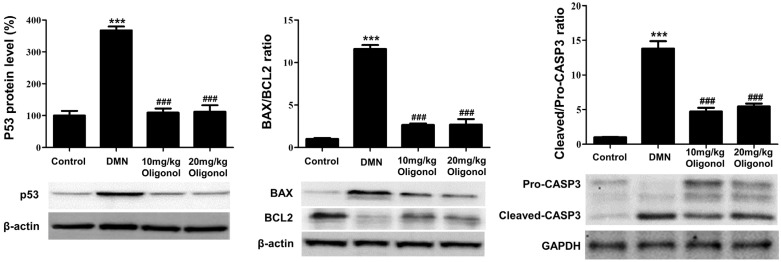
Protein levels of p53-associated apoptotic mediators perturbed by DMN and oligonol treatment. Western blot analyses of p53, BAX/BCL2 ratio, and cleaved/pro-CASP3 ratio in liver tissues from DMN and oligonol groups. Bar plots show the mean and standard error of the mean (SEM) (*n* = 3) values per group. *** *p* < 0.001 vs. the control group, and ^###^ *p* < 0.001 vs. the DMN group.

To validate the findings obtained from the transcriptomic analyses, we examined the protein levels of p53, BAX, BCL2, and CASP3 in the liver tissues of the DMN-intoxicated rats. The western blotting results showed that the protein levels of p53, along with the expression ratios of BAX/BCL2 and cleaved/pro-CASP3, were significantly increased in the livers intoxicated with DMN compared to the control group (Figure 5). In the context of apoptosis, the activated CASP3 targets PARP and cleaves it, thereby inhibiting its DNA repair function and consequently facilitating the process of programmed cell death. Thus, we additionally assessed the association between apoptosis induction and caspase-3-mediated PARP cleavage. The active form of cleaved PARP was increased in the DMN-treated rats but was effectively reduced by the oligonol treatment (Supplementary Figure S4). However, the treatment with oligonol resulted in the downregulations of p53, BAX, and cleaved CASP3 and an upregulation of BCL2 compared with those of the DMN group, albeit not in a dose-dependent manner. These results indicate that the apoptotic pathway, involving p53, BAX, and CASP3, contributed to both the liver fibrogenesis and the anti-fibrotic effects of the oligonol.

## 4. Discussion

Through analysis of the transcriptome data from the three distinct liver fibrotic models, the present study unveiled significant alterations in gene expression associated with key features of liver fibrosis (Figure 1B,C). Of particular significance is the compelling association identified between liver fibrosis and the burn-wound-healing pathway. Liver fibrosis is a pathological process that often develops in response to chronic liver injuries. This process shares many similarities with the wound-healing process, wherein the liver responds to injury by initiating a cascade of cellular events aimed at tissue repair. This parallel is particularly evident in the activations of inflammation and various cell types as well as signaling molecules. However, a critical distinction arises in liver fibrosis, where the balance between tissue production, remodeling, and degradation is disrupted, resulting in an excessive accumulation of scar tissue [44]. Furthermore, it is crucial to note that liver fibrosis has the potential to advance into cirrhosis, a condition marked by extensive scarring and compromised liver function. Intriguingly, the progression of liver fibrosis closely mirrors the pathways involved in burn-wound healing. Both liver fibrosis and burn-wound healing exhibit a common characteristic: the excessive formation of fibrotic tissue. This underscores the significance of leveraging our insights regarding burn-wound healing to enhance our comprehension of liver fibrosis and its underlying mechanisms [45].

At the core of both liver fibrosis and the burn-wound-healing process lies apoptosis, a regulated mechanism of cell death essential for the effective elimination of damaged or dysfunctional cells. In the context of chronic liver injury, hepatocytes undergo repeated cycles of death and regeneration, frequently involving apoptosis. This apoptotic event in hepatocytes serves as a trigger for the inflammatory response and the activation of hepatic stellate cells, which play a critical role in fibrosis development [46]. In the advanced stages of liver fibrosis or cirrhosis, there is a notable escalation in hepatocyte apoptosis, further exacerbating the impairment of liver function and contributing to its progressive decline [47,48]. Hence, maintaining a delicate balance of apoptosis is crucial in both burn-wound healing and liver fibrosis. Insufficient apoptosis may result in chronic inflammation and fibrosis, while excessive or inappropriate apoptosis can cause tissue damage and hinder proper healing [49]. A comprehensive understanding of apoptosis regulation in these processes holds the potential to unveil novel therapeutic targets for intervention. Apoptosis linked to liver disease is now recognized as a critical nexus where multiple key pathways intersect. Under injurious stimuli, the activation of CASP3 or proapoptotic members of the Bcl2 family (such as Bax and Bid) facilitates the release of mitochondrial cytochrome c [43,44]. Furthermore, it is noteworthy that activated CASP3 also plays a key role in the cleavage of PARP, another pivotal enzyme in the process of apoptosis. PARP, when cleaved by CASP3, loses its ability to repair DNA and maintain cell survival, thereby pushing the cell toward apoptosis [50,51]. Additionally, the tumor suppressor gene *Tp53* acts as a proapoptotic factor and is triggered in response to cellular stresses, including oxidative stress [45]. The activation of the p53/Bax/Bcl2 signaling pathway intensifies cell apoptosis, thereby exacerbating the progression of liver fibrosis [46]. Building upon the findings from our transcriptome analysis, we focused our investigation on the p53 signaling pathways in a rat model of liver fibrosis induced by DMN. Remarkably, we observed substantial elevations in the p53 protein levels, along with increased BAX/BCL2 and cleaved/pro-CASP3, and larger cleaved/full length-PARP ratios in the livers of rats intoxicated with DMN (Figure 4). These pronounced increases strongly indicated the presence of apoptosis in these fibrotic livers.

Additionally, we investigated the influence of oligonol treatment on the p53 signaling pathways in the rat liver. Oligonol is a phenolic compound derived from the lychee fruit (*Litchi chinensis* Sonn.) through a manufacturing process that converts polyphenol polymers into oligomers [52]. This powerful anti-oxidant supplement has shown diverse physiological and biochemical effects in different in vivo models [53,54,55]. In a prior study, we uncovered that oligonol possesses anti-fibrotic qualities in combating DMN-induced rat liver fibrosis. Its anti-fibrotic activity largely stems from its ability to mitigate HSC activation [16]. It achieves this by reducing hepatic inflammation through the inhibition of NF-κB activation during the initial stages of liver fibrosis and by restoring the oxidative balance through Nrf2 activation. This dual action helps prevent lipid peroxidation and the production of ROSs, both of which are key factors in the development of liver fibrosis. In the present study, we have demonstrated that the administration of oligonol to DMN-intoxicated rats led to the downregulations of various genes associated with p53 transcription (Figure 2E) and effectively reversed the overexpressions of crucial apoptotic genes, including *Bax, Tp53*, and *Casp3* (Figure 2E and Figure 4). These findings underscore the crucial role of the apoptosis pathway in liver fibrosis and emphasize the importance of suppressing this pathway to achieve anti-fibrotic effects. However, this study predominantly targeted a broad analysis of liver tissue and did not delve into a comprehensive examination to ascertain whether apoptosis was more prevalent in specific cell types. Thus, the relationship between the detected apoptotic signature and stem cell exhaustion, along with the distribution of apoptosis among diverse liver cell populations, indeed necessitates further investigation. Moreover, it is essential to acknowledge that the apoptosis of activated HSCs is a critical mechanism in the resolution of liver fibrosis [56]. When these cells escape apoptosis, they continue to produce excessive ECM amounts, leading to the accumulation of scar tissue.

While our integrative transcriptome analysis and western blot results for liver tissues have indicated that apoptosis is a prominent feature in chronically damaged liver tissue, the specific impact of this damage on the apoptosis of activated HSCs during the fibrotic process and its potential prevention are still areas requiring further investigation. This complex interplay necessitates additional exploration to fully understand the role and potential manipulation of apoptosis in effectively managing liver fibrosis. Additionally, we utilized three fibrosis models, employing male rats aged 6 to 9 weeks, in our study. A significant body of past research has utilized male rats, and in order to maintain consistency and ensure comparability with these studies, we followed this convention. This choice was made to control for any potential age-related variations in fibrosis expression. We acknowledge that this strict age range may have limited the broader applicability of our results, especially considering the increased prevalence of fibrosis in older populations. As such, the findings of this study could benefit from validation by additional studies involving female and/or older rat populations. We strongly encourage further research to explore whether our findings can be generalized to these different groups, thus providing a more comprehensive understanding of liver fibrosis progression and the associated mechanisms.

## 5. Conclusions

**Figure 6:**
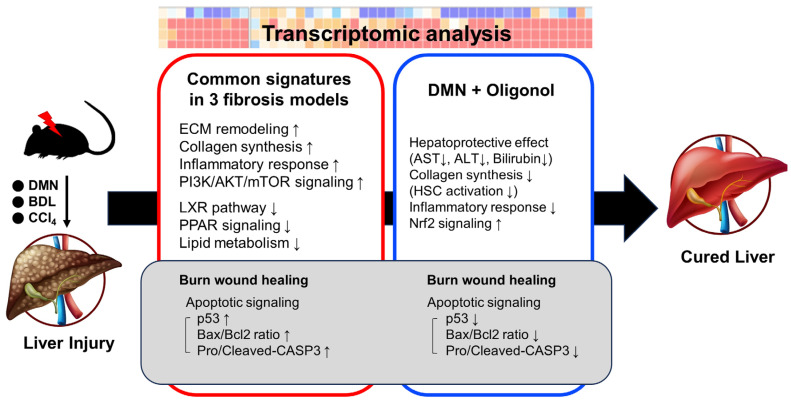
A summary illustration of the findings of this study.

By conducting an integrative transcriptomic analysis across three distinct fibrotic rat models, we have illuminated compelling insights into the molecular alterations inherent in liver fibrosis and uncovered upregulated apoptotic signaling within the wound-healing pathway in liver fibrosis models (Figure 6). Our findings suggest that modulation of this pathway, specifically the balance of apoptosis, could potentially serve as a therapeutic strategy for managing liver fibrosis. Additionally, our study has exhibited the potential of oligonol, a lychee-derived phenolic compound, in counteracting the overexpression of critical apoptotic genes in a DMN-induced rat model. These results point towards the potential therapeutic application of oligonol in conditions marked by excessive fibrosis, such as liver fibrosis.

However, the exact mechanisms through which oligonol operates and its impact on the apoptosis of activated HSCs during the fibrotic process still need to be defined. Furthermore, while oligonol may alleviate DMN-induced liver fibrosis by inhibiting p53-mediated apoptosis, there is a potential risk that it could amplify the likelihood of liver cancer if it overly suppresses the p53 pathway. Thus, while exploring the modulation of the p53 pathway with oligonol has presented a promising avenue in liver disease research, the intertwined roles of p53 in both liver fibrosis and cancer mandate a judicious and knowledgeable approach. Our findings have underscored the need for further investigations to build upon this initial research and delve deeper into the underlying mechanisms involved in liver fibrosis and its treatment.

## Figures and Tables

**Table 1:** Details of the three liver fibrosis models utilized in this study.

Biological Samples	Source	Animal/Sex/Age	Sequencing Platform	No. of Samples (Per Group)
Livers from DMN Model	This study	SD rat/male/6~7 weeks	DNBSEQ-T7	3 (1 normal, 1 fibrosis, 1 oligonol)
Livers from BDL Model	GEO (GSE152250, [22])	SD rat/male/6~8 weeks	HiSeq 4000	11 (6 normal, 5 fibrosis)
Livers from CCl_4_ Model	GEO (GSE189402, [23])	SD rat/male/8 weeks	NovaSeq 6000	6 (3 normal, 3 fibrosis)

GEO, Gene Expression Omnibus; DMN, dimethylnitrosamine; CCl_4_, carbon tetrachloride; BDL, bile duct ligation.

## Data Availability

The authors confirm that the data supporting the findings of this study are available upon request.

## Supplementary Materials

The following supporting information can be downloaded at: https://www.mdpi.com/article/10.3390/antiox12081588/s1, Figure S1: Liver gene expression signatures of multiple liver fibrosis rat models; Figure S2: Transcriptomic changes by oligonol administration compared to liver fibrosis models; Figure S3: Correlations in p53-related genes among multiple fibrosis models; Figure S4: Activation of CASP3-mediated PARP cleavage induced by DMN and oligonol treatment; Table S1: Upregulated and downregulated genes in DMN-induced liver fibrosis model; Table S2: Upregulated and downregulated genes in BDL-induced liver fibrosis model; Table S3: Upregulated and downregulated genes in CCl4-induced liver fibrosis model; Table S4: Top 10 pathways enriched for commonly upregulated or downregulated DEGs in three liver fibrotic models; Table S5: Upregulated and downregulated genes in oligonol-administered liver fibrosis; Table S6: Top ten upregulated and downregulated pathways in oligonol-treated DMN model vs. untreated DMN model.
