# Supplementary material for: Integrative Transcriptomic Analysis Reveals Upregulated Apoptotic Signaling in Wound-Healing Pathway in Rat Liver Fibrosis Models
Source: Antioxidants (Basel). 2023 Aug 9;12(8):1588. doi: 10.3390/antiox12081588 (PMC10451232; doi:10.3390/antiox12081588)
Supplement: Supplementary file 1 [file antioxidants-12-01588-s001.zip › antioxidants-2481078-supplementary figures.pdf]

Supplementary Figure S1

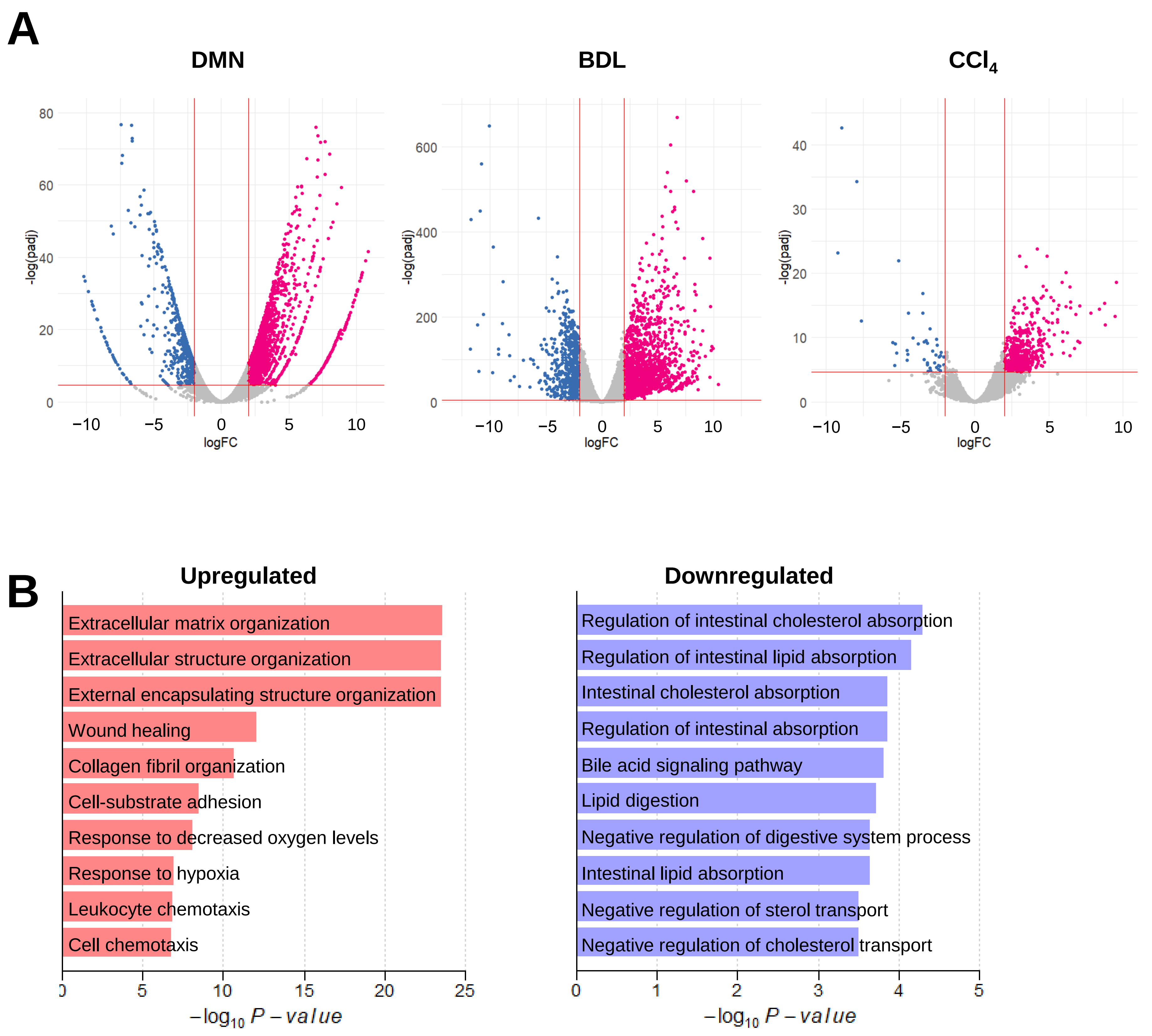

Supplementary Figure S1. Liver gene expression signatures of multiple liver fibrosis rat models. (A) Volcano plots showing gene expression changes in liver tissues of DMN-, BDL-, and CCl_4-induced liver fibrosis rat models compared to normal liver tissues. Differentially expressed genes (DEGs) were selected based on the following cut-offs: false discovery rate (FDR)-adjusted p-value < 0.01 and $|\log_2\text{fold-change}| > \log_2(2)$. (B) Top ten Gene ontology (GO) terms significantly enriched in upregulated and downregulated DEGs in common across all three liver fibrosis models. DMN, dimethylnitrosamine; BDL, bile duct ligation; CCl_4, carbon tetrachloride.

Supplementary Figure S2

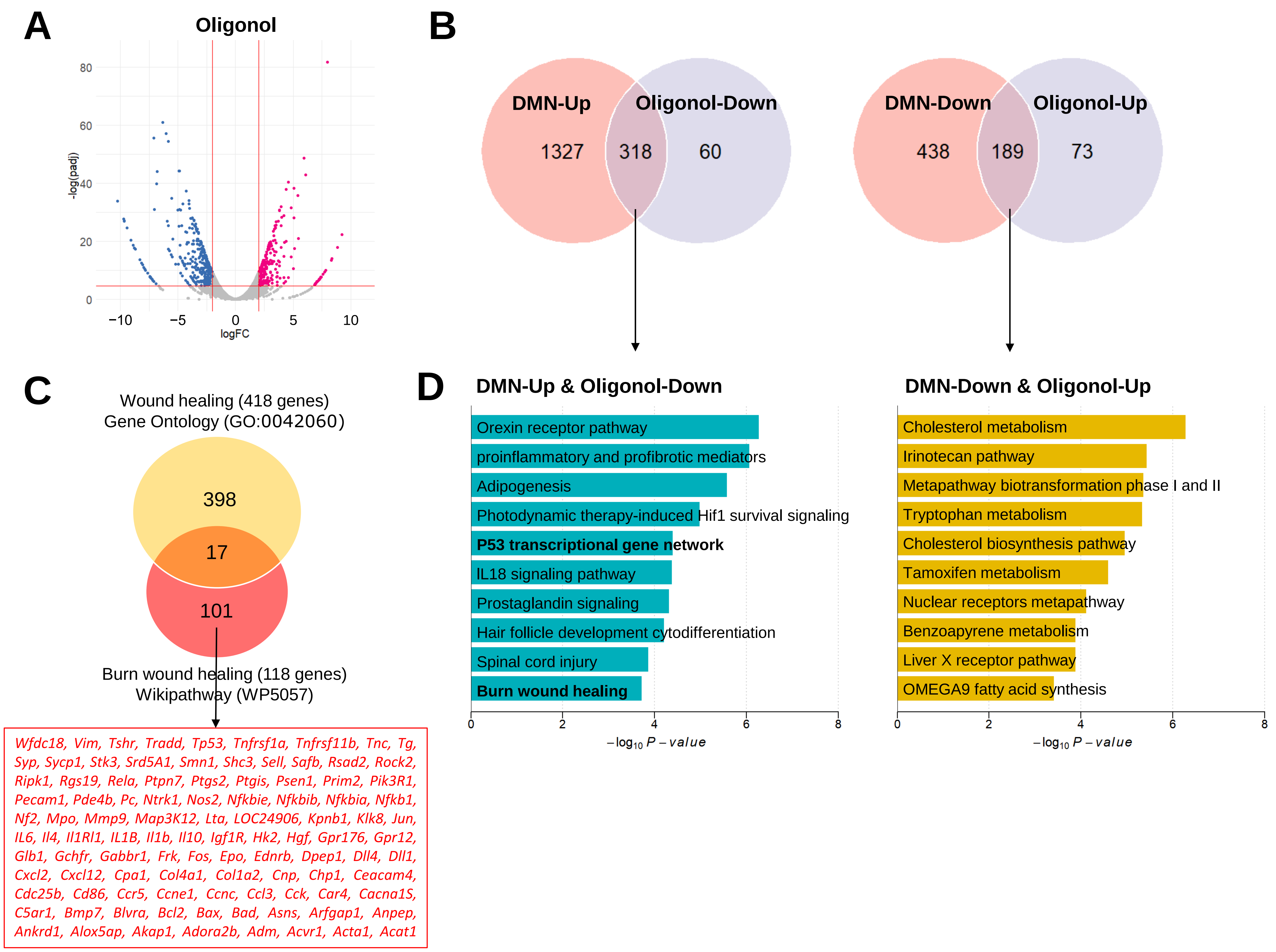

Transcriptomic changes by oligonol administration compared to DMN-induced fibrosis models. (A) Volcano plots showing gene expression changes in liver tissues of oligonol treated DMN-liver fibrosis models compared to untreated DMN-liver fibrosis models . Differentially expressed genes (DEGs) were selected based on the following cut-offs: false discovery rate (FDR)-adjusted p-value < 0.01 and $|\log_2$fold-change$| > \log_2(2)$. (B) Venn diagrams showing the overlap of DEGs of DMN-liver fibrosis model and oligonol treated DMN-liver fibrosis model. (C) Overlap of genes involved in the two gene sets: wound healing and burn wound healing. Wound healing genes were obtained from Gene ontology (GO) databases (GO:0040260) and the burn wound healing genes were obtained from Wikipathways (WP5057) via MSigDB and Cytoscape App ‘Wikipathways’. (D) Top ten significantly enriched pathways for 318 DEGs (left) and 189 DEGs (right).

Supplementary Figure S3

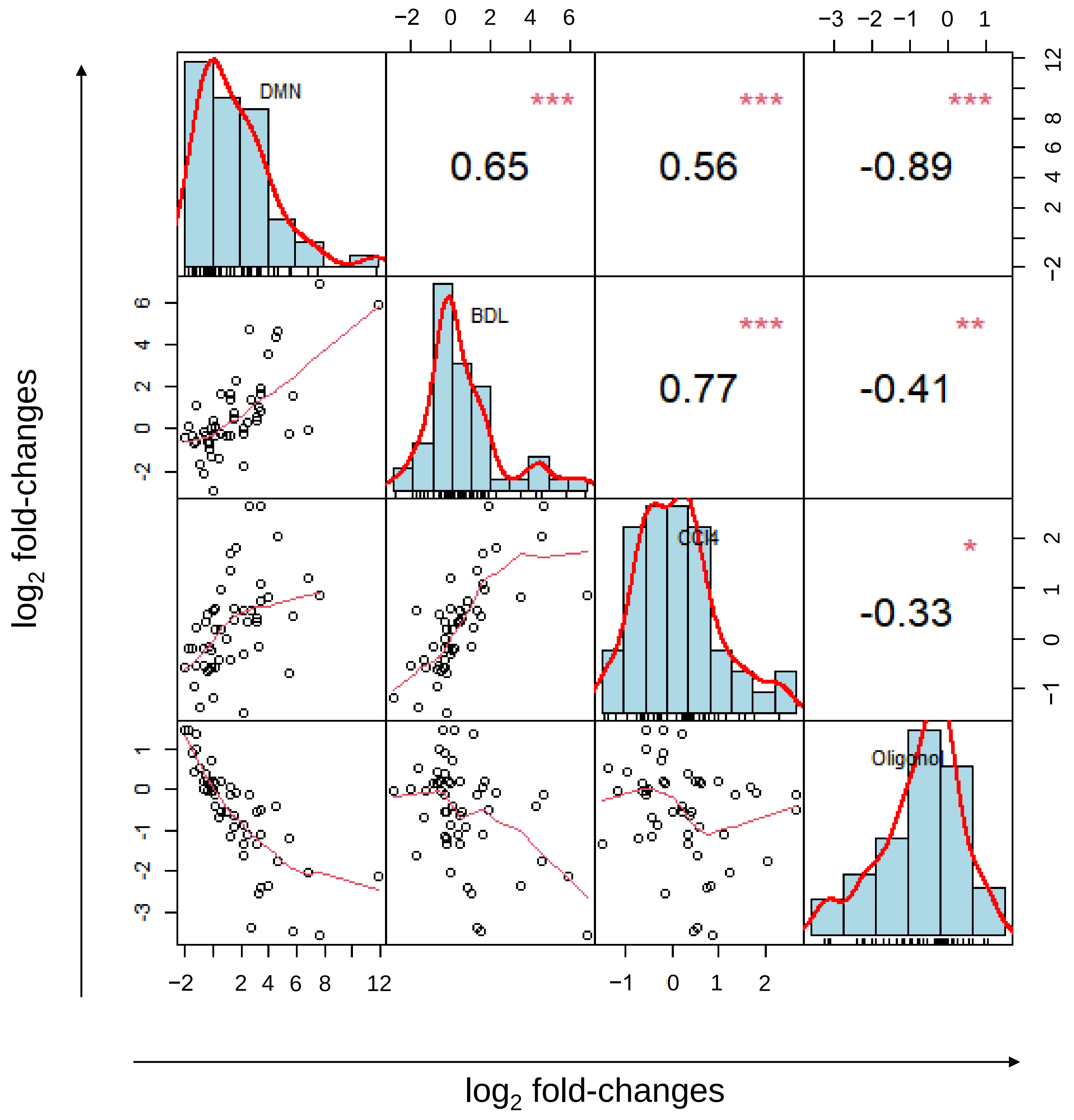

Correlations in p53-related genes among multiple fibrosis models. Association of log2 fold-changes of genes involved in the gene set ‘p53 transcriptional gene network’ among liver tissues from three liver fibrosis models (DMN, BDL, and CCI4) and oligonol-treated DMN model. *, $p < 0.05$; **, $p < 0.01$; ***, $p < 0.001$.

Supplementary Figure S4

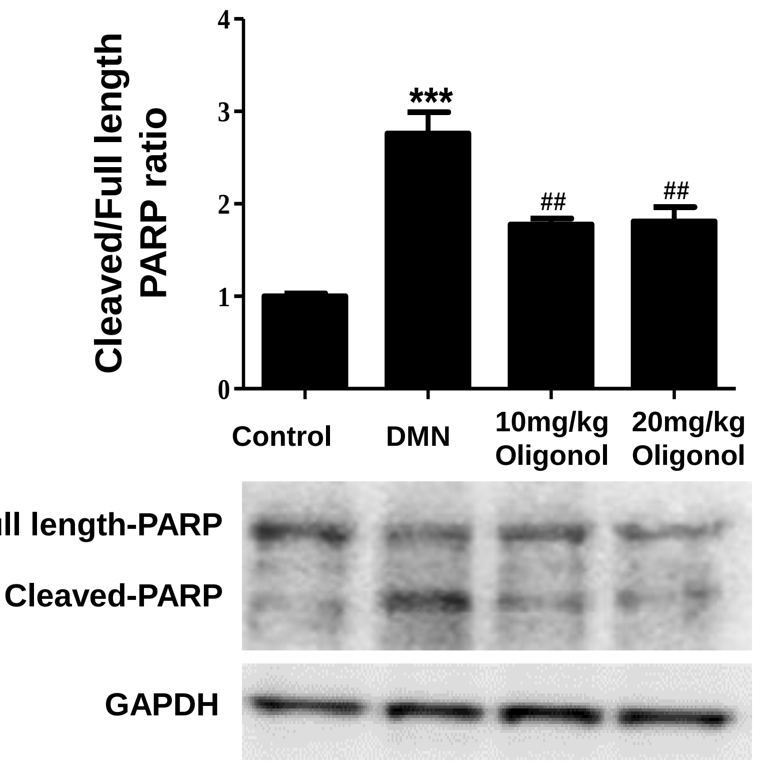

Activation of CASP3-mediated PARP cleavage induced by DMN and oligonol treatment.

Western blot analysis of cleaved/full length PARP ratio in liver tissue from DMN and oligonol groups. Barplots show the mean and standard error of the mean (S.E.M) (n=3) values per group. *** $p < 0.001$ vs. the control group, ## $p < 0.01$ vs. the DMN group.
